# Pregnancy-induced gait alterations: meta-regression evidence of spatiotemporal adjustments

**DOI:** 10.3389/fbioe.2024.1506002

**Published:** 2024-12-17

**Authors:** Xin Li, Zhenghui Lu, Yang Song, Minjun Liang, Yi Yuan, Gusztáv Fekete, András Kovács, Dong Sun, Yaodong Gu

**Affiliations:** ^1^ Research Academy of Medicine Combining Sports, Ningbo, China; ^2^ Faculty of Engineering, University of Pannonia, Veszprém, Hungary; ^3^ Department of Biomedical Engineering, Faculty of Engineering, The Hong Kong Polytechnic University, Hong Kong SAR, China; ^4^ Faculty of Sports Science, Ningbo University, Ningbo, China; ^5^ Department of Material Science and Technology, AUDI Hungária Faculty of Vehicle Engineering, Széchenyi István University, Győr, Hungary

**Keywords:** pregnancy, gait, spatiotemporal gait parameters, biomechanical changes, Metaregression

## Abstract

During pregnancy, women undergo significant physiological, hormonal, and biomechanical changes that influence their gait. The forward shift of the center of mass and increased joint loads often result in a “waddling gait,” elevating the risk of falls. While gait changes during pregnancy have been documented, findings across studies remain inconsistent, particularly regarding variations at different pregnancy stages. This systematic review and meta-analysis aimed to quantify the impact of pregnancy stages on spatiotemporal gait parameters. A comprehensive literature search across six databases (PubMed, Web of Science, Scopus, EBSCO, Embase, and Cochrane Library) was conducted to identify studies on pregnancy and gait, and data on publication details, methodology, participant characteristics, gait outcomes, and study limitations were extracted. Out of 4,581 initial records, 21 studies met the inclusion criteria. The meta-analysis revealed significant changes in gait parameters during pregnancy, with decreases in stride length (effect size = −0.29) and gait speed (effect size = −0.55), and increases in stride width (effect size = 0.45), cycle time (effect size = 0.38), and double support time (effect size = 0.41). Meta-regression analyses indicated that gestational weeks significantly impacted stride length (β = −0.03 [95% CI, −0.055 to −0.002], p < 0.05) and stride width (β = 0.02 [95% CI, 0.003 to 0.039], p < 0.05), while no significant effects were found for cycle time, double support time, or gait speed. In conclusion, pregnancy leads to significant changes in gait patterns, with a notable increase in stride width and a decrease in stride length as gestation progresses, suggesting these adjustments are strategies for maintaining balance and stability in response to physiological changes. The analysis also emphasizes that while gestational age influences gait adaptations, other factors such as pelvic girdle pain, footwear, and psychological influences play crucial roles. Understanding these complex gait changes can inform interventions and guidelines to support mobility and safety for pregnant women throughout their pregnancy.

## 1 Introduction

As pregnancy progresses, a woman’s body experiences a series of rapid adaptations to support the growth and development of the fetus, including physiological (Kazma et al., 2020), hormonal (Feldt–Rasmussen and Mathiesen, 2011), emotional (Ibrahim et al., 2019), and biomechanical adaptations (Wong and McGregor, 2018; Zia et al., 2022). Physiologically, pregnancy involves increased plasma volume, cardiovascular changes, and metabolic adjustments (Kazma et al., 2020; Aguree and Gernand, 2019). Hormonal changes, such as elevated progesterone and relaxin, loosen ligaments and affect muscle tone in preparation for childbirth, which can also contribute to emotional fluctuations and increased sensitivity, potentially influencing gait and mobility (Murray and Hendley, 2020; Atay and Iz, 2015). Emotional fluctuations during pregnancy, driven by both hormonal shifts and the psychological demands of impending motherhood, may lead to increased stress levels that affect physical health behaviors such as exercise and mobility, which in turn can influence gait stability and overall movement patterns (Ibrahim et al., 2019; Bjelica et al., 2018). Biomechanically, the most noticeable change is the forward and downward shift of the body’s center of mass due to increased mass in the anterior lower trunk, leading to altered moment arms around the hip joint and an increase in hip adduction moments (Foti et al., 2000). Notably, these biomechanical changes can persist up to 5 years *postpartum* (Bertuit et al., 2015; Fukan et al., 2024).

Walking, as one of the most common physical activities during pregnancy (Nascimento et al., 2015), is greatly affected by these biomechanical changes, resulting in unique spatiotemporal gait characteristics (Wong and McGregor, 2018). The postural and gait adjustments during pregnancy are often characterized by increased lateral sway and altered pelvic movement, which have been linked to an elevated risk of instability and falling incidents (Foti et al., 2000; Mei et al., 2018; Sunaga et al., 2013; Branco et al., 2022; Forczek et al., 2020; McCrory et al., 2014).

Various studies have documented that pregnancy leads to changes in gait parameters, such as reduced walking speed and cadence, shortened step length (Zia et al., 2022; Bertuit et al., 2015; Takeda et al., 2009; Carpes et al., 2008; Branco et al., 2013), increased double support time (Carpes et al., 2008; Branco et al., 2013; Ramachandra, 2018), and a wider stance (Branco et al., 2013; Forczek and Staszkiewicz, 2012; Christensen et al., 2019; Krkeljas, 2018). Additionally, compensation through the mediolateral component of the ground reaction force has been observed (Forczek et al., 2018). However, Foti et al. (2000) offered a different perspective, suggesting that gait parameters can be adjusted by increasing the load on hip and ankle muscles to maintain gait stability.

The impact of pregnancy on walking may vary across different stages, but treating all pregnant women as a single group overlooks these trimester-specific variations. Previous studies attempting to quantify these differences have been limited by small sample sizes (Bertuit et al., 2015; Forczek et al., 2020; Branco et al., 2013; Ramachandra, 2018; Krkeljas, 2018; Gimunová et al., 2020; Forczek et al., 2019; ElDeeb et al., 2016; Błaszczyk et al., 2016). Meta-analysis offers a way to address this limitation by synthesizing data across multiple studies, providing a clearer picture of trimester-specific gait changes.

This study seeks to perform a systematic review and meta-analysis to measure the extent of the impact of pregnancy on spatiotemporal gait characteristics and to explore dynamic trends across different pregnancy stages. We hypothesize that a linear relationship exists between gestational weeks and gait parameters, drawing on prior research that suggests progressive gait adjustments with advancing pregnancy stages (Gilleard, 2013). This study aims to test this hypothesis, offering further insights into how pregnancy progression affects gait changes.

## 2 Methods

This systematic review was conducted in accordance with the Preferred Reporting Items for Systematic Reviews and Meta-Analysis (PRISMA) Statement (Moher et al., 2015) and Meta-Analysis of Observational Studies in Epidemiology (MOOSE) guidelines (Stroup et al., 2000). The review protocol was registered with PROSPERO (Registration No. CRD42023423741).

### 2.1 Eligibility criteria

The inclusion criteria for the studies were defined using the PICO (Population, Intervention, Comparison, and Outcomes) framework, as outlined in Table 1. The population consisted of healthy pregnant participants at clearly defined stages of pregnancy, specifically excluding those with high-risk pregnancies or any pre-existing medical conditions that could influence gait, such as musculoskeletal disorders, hypertension, or diabetes. The pregnancy period is categorized into three distinct trimesters: the first trimester, spanning from conception to 13 weeks; the second trimester, covering weeks 14–27; and the third trimester, extending from 28 to 40 weeks. The intervention focused on walking at a self-selected pace, while comparison groups included non-pregnant participants, *postpartum* women, or those in early pregnancy (under 12 weeks). The primary outcomes assessed were spatiotemporal gait parameters. Studies were excluded if they were abstracts, review articles, or case studies. In cases where multiple studies involved the same participant sample, only the primary study was included to avoid duplication.

**TABLE 1:** Inclusion criteria following the PICO framework.

Criteria	Characteristics
Population	Healthy pregnant women, excludes high-risk pregnancies or gait-affecting conditions (e.g., musculoskeletal disorders, hypertension, diabetes)
Intervention	Walking at a self-selected speed
Comparison	Non-pregnant participants, *postpartum* women, or those in early pregnancy (under 12 weeks)
Outcomes	Spatiotemporal gait parameters

### 2.2 Search strategy and study selection

Two reviewers working independently (LX and ZH) conducted an extensive literature search was conducted using six databases, including PubMed, Web of Science, Scopus, EBSCO, Embase, and the Cochrane Library. The initial search was conducted in April 2023 and updated in February 2024, with no additional studies identified in the updated search. The complete search strategy is detailed in Appendix 1, and no date restrictions were applied to ensure comprehensive coverage of relevant literature. A total of 4,581 articles were retrieved, which were imported into EndNote X9 (Clarivate, Philadelphia, Pennsylvania, United States) for management, and 1,838 duplicates were systematically removed.

**FIGURE 1:**
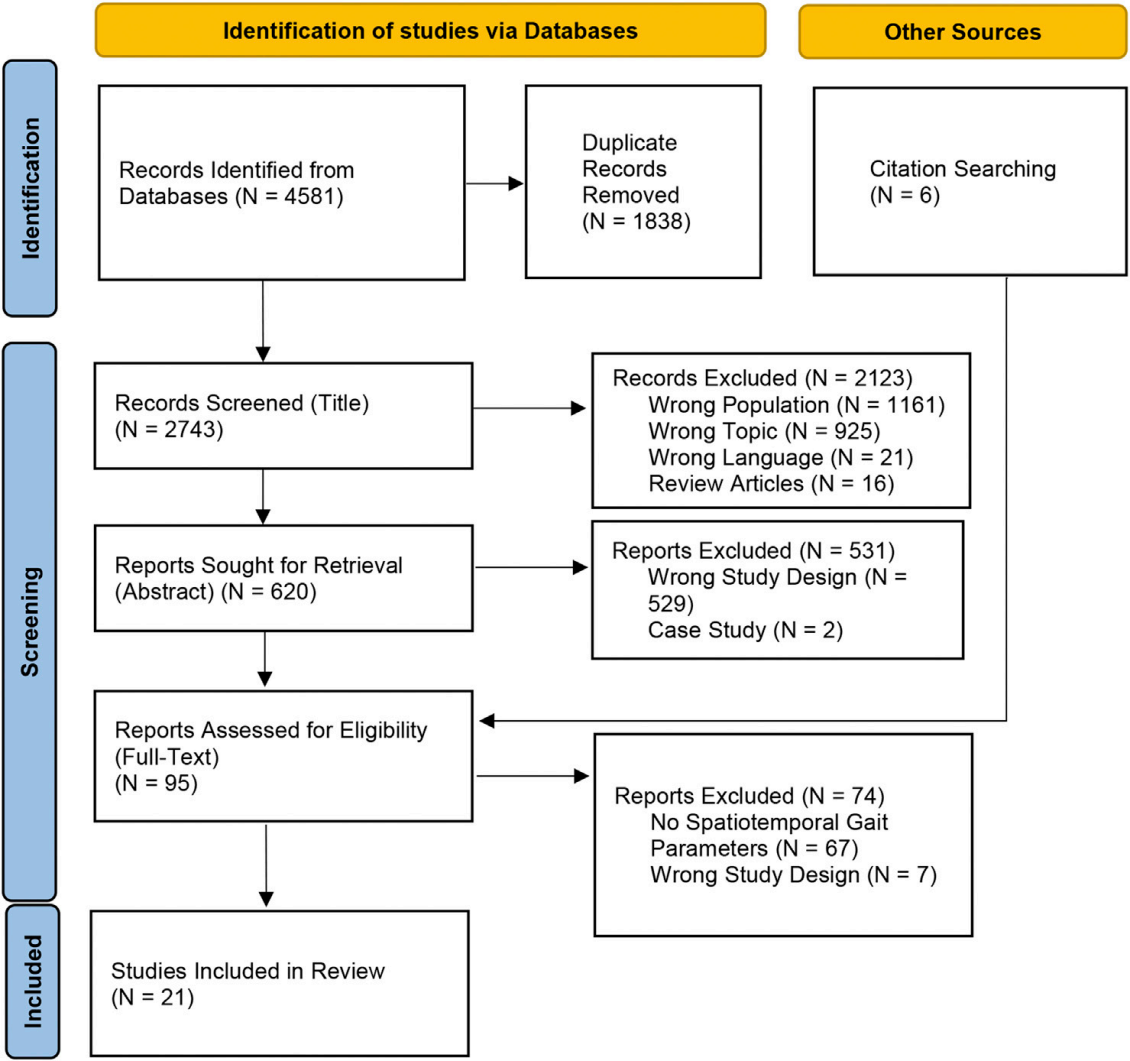
PRISMA flow diagram illustrating the study selection process for the systematic review (N = 21).

Following duplicate removal, 2,743 articles remained for screening. Two reviewers (LX and SY) independently evaluated these articles based on their titles and abstracts, resulting in the exclusion of 2,654 articles that did not meet the eligibility criteria. An additional six articles were identified through a manual review of reference lists from relevant studies. In total, 95 full-text articles were assessed for eligibility, of which 74 were excluded for various reasons. Ultimately, 21 studies met the inclusion criteria and were included in the qualitative and quantitative analyses, demonstrating a high level of inter-rater reliability (kappa = 0.87, 95% CI: 0.70–1.00) (Landis and Koch, 1977). The detailed study selection process is illustrated in the PRISMA flow diagram (Figure 1) (Page et al., 2021).

### 2.3 Quality assessment and data extraction

The risk of bias for each included study was assessed using the QualSyst critical appraisal tool for quantitative research (Kmet et al., 2004). QualSyst was selected due to its comprehensive evaluation of methodological rigor, making it well-suited for assessing the quality of diverse quantitative studies. This tool evaluates 14 specific criteria (outlined in Table 2), which cover aspects such as study design, data collection methods, and statistical analysis. Each criterion is scored as follows: “yes” = 2, “partial” = 1, “no” = 0, and “not applicable” = “NA.” Two independent reviewers (LX and ZH) conducted the quality assessments to ensure objectivity and minimize bias, with any disagreements resolved by a third reviewer (SY) to enhance reliability. Based on the total scores, studies were categorized as high quality (≥75%), moderate quality (55%–74%), or low quality (<55%). This thorough evaluation guaranteed that only high-quality studies were included, strengthening the credibility of the review’s results.

**TABLE 2:** Quality assessment ‘Qualsyst’.

Study	Question	Study design	Subject selection	Characteristics	Randomization	Blinded researchers	Blinded subjects	Outcome	Sample size	Analysis	Variance	Confounding	Results detail	Conclusion	Rating (%)
Zia et al. (2022)	2	2	1	2	0	0	0	2	2	1	1	1	2	2	81.82%
Gimunová et al. (2020)	2	2	1	2	1	0	0	2	2	2	1	1	2	2	83.33%
Bagwell et al. (2020)	2	2	1	2	0	0	0	2	2	2	1	1	2	2	86.36%
Forczek et al. (2019)	2	2	1	2	0	0	0	2	2	2	1	1	2	2	86.36%
Christensen et al. (2019)	2	2	1	2	0	0	0	2	2	2	1	1	2	2	86.36%
Ramachandra (2018)	2	2	1	2	0	0	0	2	2	2	1	1	2	2	86.36%
Krkeljas (2018)	2	2	1	2	0	0	0	2	2	2	1	1	2	2	86.36%
Kerbourc’h et al. (2017)	2	2	1	2	0	0	0	2	2	2	1	1	2	2	86.36%
ElDeeb et al. (2016)	2	2	1	2	0	0	0	2	2	2	1	1	2	2	86.36%
Błaszczyk et al. (2016)	2	2	1	2	0	0	0	2	2	2	1	1	2	2	86.36%
Branco et al. (2013)	2	2	1	2	0	0	0	2	2	2	1	1	2	2	86.36%
Lymbery and Gilleard (2005)	2	2	1	2	0	0	0	2	2	2	2	1	2	2	90.91%
Bird et al. (1999)	2	2	1	2	0	0	0	2	2	2	2	1	2	2	90.91%
Branco et al. (2016)	2	2	1	2	0	0	0	2	2	2	1	1	2	2	86.36%
Foti et al. (2000)	2	2	1	2	0	0	0	2	2	2	1	1	2	2	86.36%
Bertuit et al. (2015)	2	2	1	2	0	0	0	2	2	2	1	1	2	2	86.36%
Carpes et al. (2008)	2	2	1	2	0	0	0	2	1	2	1	1	2	2	81.82%
Forczek and Staszkiewicz (2012)	2	2	1	2	0	0	0	2	2	2	1	1	2	2	86.36%
Wu et al. (2010)	2	2	1	2	0	0	0	2	1	2	1	1	2	2	81.82%
Gilleard (2013)	2	2	1	2	0	0	0	2	2	2	1	1	2	2	86.36%
Rothwell et al. (2020)	2	2	2	1	0	0	0	2	2	2	1	1	2	2	86.36%

Scoring: 2 = Yes, 1 = Partial, 0 = Not applicable; Quality levels: ≥75% High, 55%–74% Moderate, ≤55% Low.

For the quantitative synthesis, the means, medians, and standard deviations were collected from the results sections and tables of the included manuscripts. When necessary, the Web Plot Digitizer program was used to extract missing data. If data were unavailable or incomplete, the corresponding authors were contacted. Data extraction was conducted by one reviewer (LX) and independently verified by another (MJ), with any discrepancies resolved through consensus. Tables 3, 4 provide an overview of participant characteristics and methodological details of the studies. The extracted data encompassed study details (including study ID and country), the stage of pregnancy, sample size, participant characteristics such as age (years), BMI (kg/m^2^), and other relevant traits, the study design, footwear conditions, the type of system used for gait analysis, the walking protocol, outcome measures, and any study limitations.

**TABLE 3:** Study and participant characteristics overview.

Study	Country	Pregnancy stage	Sample size	Age (years)	BMI (kg/m^2^)	Participant characteristics
Zia et al. (2022)	Pakistan	NP 3rd trim	141 69	27.4 ± 4.34 26.3 ± 5.15	NA NA	24.6% primigravida, 17.4% secundigravida, 26% tertigravida, 31.9% multigravida; 28% nulliparous, 31% primiparous, 17.4% biparous/multiparous, 4.3% multipara
Gimunová et al. (2020)	Czech	14 w.p 28 w.p 37 w.p 28 w. Postp	18 18 18 18	Started 28.94 ± 3.22 32.25 ± 3.43	22.8 ± 4.11 25.5 ± 4.45 27.2 ± 4.64 22.5 ± 4.22	Excluding movement limitations and twin/triplet pregnancies
Bagwell et al. (2020)	United States	2nd trim 3rd trim NP	23 22 20	31.6 ± 3.4 31.6 ± 3.4 32.1 ± 4.7	26.3 ± 4.35 28.3 ± 4.49 23.7 ± 4.53	Excluded history of back surgery or exercise contraindications
Forczek et al. (2019)	Poland	1st trim 2nd trim 3rd trim	36 30 30	Started 30.3 ± 3.4	21.9 ± 2.0 24.0 ± 2.2 25.9 ± 2.7	Excluded prior orthopedic/neurological injuries; 19 primigravid, 8 in second pregnancy, 3 in third
Christensen et al. (2019)	Norway	NP 2nd trim	24 24	31.4 ± 4.0 31.5 ± 3.7	24.0 ± 3.29 23.0 ± 2.85	NA
Ramachandra et al. (2018)	India	1st trim 2nd trim 3rd trim 6 days. Postp 32w. Postp	70 70 70 70 70	Started 27.98 ± 3.65	23.08 ± 4.17 NA NA 25.67 ± 4.94 NA	Primigravidae; excluded history of musculoskeletal issues, flat feet, limb/back pain, hydramnios, and fibroids
Krkeljas (2018)	South Africa	1st trim 2nd trim 3rd trim	14 20 10	28.1 ± 5.5 27.1 ± 6.1 26.6 ± 6.6	24.3 ± 4.0 27.7 ± 6.2 29.9 ± 4.9	Participants who were high-risk, unable to complete the test, or had physical/musculoskeletal limitations were excluded
Kerbourc’h et al. (2017)	Belgium	2nd trim NP	61 22	29 ± 5 27 ± 5	27 ± 5 22 ± 3	Excluded lumbopelvic pain and sacroiliac/pubis pain during pregnancy
Eldeeb et al. (2016)	Egypt	1st trim 2nd trim 3rd trim	20 20 20	Started was 24 ± 2 years	23.21 ± 1.90	Women with diabetes, preeclampsia, multiple pregnancies, back, sacroiliac, or symphyseal pain, cardiac or neurological conditions, deformities, or a history of back or limb surgeries were excluded
Błaszczyk et al. (2016)	Poland	1st trim 3rd trim 8w. Postp 24w. Postp	28 28 28 28	Started 28.2 ± 3.4 years	22 ± 2.5 26.6 ± 3.0 23.1 ± 2.8 22.3 ± 2.7	Participants had no history of musculoskeletal or neurological abnormalities, uncorrectable vision problems, obesity, or any conditions that could affect walking
Branco et al. (2013)	Portugal	2nd trim 3rd trim NP	22 22 22	Started 32.5 ± 2.6	25.6 ± 2.9 27.3 ± 2.8 21.5 ± 2.4	Participants had no history of trauma or disease affecting the foot, ankle, knee, musculoskeletal, or neuromuscular systems
Lymbery and Gilleard (2005)	Australia	3rd trim 8 w. Postp	13 13	Started 27.8 ± 1.2	27.28 ± 5.67 23.54 ± 5.20	Included primiparous/multiparous women with a single fetus; excluded those with above-average pre-pregnancy height-weight, multiple fetuses, or musculoskeletal issues affecting gait in the past 6 months
Bird et al. (1999)	Australia	1st trim 3rd trim	34 25	Started 28.7 ± 5.3	Started 25.14 ± 6.07	NA
Branco et al. (2016)	Portugal	1st trim 2nd trim 3rd trim Postp	11 11 11 11	Started 33.20 ± 1.62	22.7 ± 2.7 24.7 ± 3.6 26.4 ± 3.4 23.2 ± 3.3	No history of trauma/disease in foot, ankle, knee, musculoskeletal, or neuromuscular
Foti et al. (2000)	US	3rd trim 1 year. Postp	15 15	1.67	22.63 ± 1.58 27.29 ± 1.62	NA
Bertuit et al. (2015)	Belgium	24 w.p 28 w.p 32 w.p 36 w.p NP	8 17 23 10 23	26 ± 1 28 ± 5 30 ± 6 29 ± 3 27 ± 5	25 ± 3 26 ± 3 28 ± 6 26 ± 3 22 ± 3	71% were childless, 27% had one child, 2% had two or more; Participants had no history of foot, ankle, or knee pain, pelvic girdle pain, neuromuscular trauma or disease, or cardiovascular conditions
Carpes et al. (2008)	Brazil	2nd trim 3rd trim 16 w postp	7 7 7	From 23 to 35	NA	NA
Forczek and Staszkiewicz (2012)	Poland	NP 3rd trim Postp	13 13 13	Started 29.15 ± 3.5	19.7 24.6 21.7	Excluded subjects with prior orthopedic or neurological injuries
Wu et al. (2010)	China	2nd trim 3rd trim NP	6 6 13	31.83 ± 3.54 34.33 ± 3.01 26.8 ± 4.4	25.55 ± 3.26 26.57 ± 2.02 22.40 ± 4.84	Primiparous/multiparous women without low back pain or gait-affecting neurological, musculoskeletal, or obstetric disorders. Excluded: lumbar spine/pelvis/hip/knee surgery, fractures, tumors, inflammation, Conditions such as Bechterew’s syndrome, Scheuermann’s syndrome, active polyarthritis, rheumatoid arthritis, severe osteoporosis, hormone-induced pregnancy, or conception via *in vitro* fertilization (IVF) were excluded
Gilleard (2013)	Australia	24 w.p 32 w.p 38 w.p NP	9 9 9 12	Started 32.6 ± 4.3 NP28.9 ± 4.1	Started 28.66 ± 4.68 NP 22.74 ± 3.02	Included 5 primigravidas and 4 multigravidas
Rothwell et al. (2020)	US	16 w.p 40 w.p 28 w postp	17 17 17	22 to 37	NA	No balance-impacting injuries/neurological conditions or high-risk pregnancies

NP, Non Pregnant women, trim. – trimester of pregnancy, w.p. – week of pregnancy, m–month, postp. – *postpartum*, NA, Not Available.

**TABLE 4:** Summary of study designs and methodological characteristics.

Study	Design	Footwear	System type	Walking protocol	Outcome measures	Limitations
Zia et al. (2022)	Cross-sectional	Barefoot	Footprints	A firm surface	Step Length ↓, Stride Length ↓, Speed ↓	N/A
Gimunová et al. (2020)	Longitudinal	Barefoot	3D motion analysis	6-m walkway Third trial	Step Cycle Time	Baseline differences, small sample size, high variability
Bagwell et al. (2020)	Longitudinal and cross-sectional	N/A	3D motion analysis	16-m walkway Seven trials	Velocity	Heterogeneity of pregnant; lack of longitudinal gait assessment
Forczek et al. (2019)	Longitudinal	Barefoot	3D motion analysis	12-m walkway 50 m	Velocity, Cadence, Single Support (2nd Trim ↓), Stride Length	N/A
Christensen et al. (2019)	Cross-sectional	Barefoot	3D motion analysis	15 m walk-way	Speed, Stride Width, Stride Length, Ipsilateral Step Length, Contralateral Step Length ↓, Cycle Time, Stance Time ↓, Stance Phase ↓, Double Limb Support ↓	Cross-sectional design, dependencies in the data, Soft tissue errors and marker validity
Ramachandra (2018)	Longitudinal	Barefoot	Gait platform	10 m pathway 3-step gait protocol	Step Duration, Double Stance Duration, Swing Duration, Step Length, Gait Cycle Length, Cadence	N/A
Krkeljas (2018)	Longitudinal	N/A	3D motion analysis	15-m walkway Three trials	Speed, Stride Length, Step Width, Clearance, Double Support Time, Swing Time	Weight gain rate varied among individuals
Kerbourc’h et al. (2017)	Cross-sectional	Barefoot	Gaitrite	6.1 m walkway Nine trials	Stance Time ↑	N/A
ElDeeb et al. (2016)	Longitudinal	Barefoot	3D motion analysis	Three trials	Velocity	No control group
Błaszczyk et al. (2016)	Longitudinal	Shoes	Limb-contact signals	10 m walkway, back and forth 10 times	Velocity ↓, Stance Time ↑, Swing Time (3rd Trim ↑), Double-Support Time↑, Cadence, Stride Length↓	N/A
Branco et al. (2013)	Longitudinal	Barefoot	3D motion analysis	10 m 3 min	Velocity, Stride Width, Stride Length (3rd Trim ↓), Cycle Time, Step Time, Stance Time, Swing Time, Double Limb Support Time	N/A
Lymbery and Gilleard (2005)	Longitudinal	N/A	3D motion analysis	11-m walkway Four trials	Step Length, Velocity ↓, Stance Time, Step Width ↑, Percentage of Stance Spent in Double Support	N/A
Bird et al. (1999)	Longitudinal	Barefoot	Footprint	5 m-walkway Five-plus footprints	Base Of Gait ↑, Step Length, Stride Length	Lack Baseline, Footprints was small, without fatigue
Branco et al. (2016)	Longitudinal	Barefoot	3D motion analysis	10 m 3 min	Velocity, Stride Width, Stride Length, Step Length, Cycle Time, Step Time, Stance Time, Double Limb Support Time	N/A
Foti et al. (2000)	Longitudinal	N/A	3D motion analysis	12 m Six to eight strides	Velocity, Stride Length, Cadence, Single-Support Time ↓, Double-Support Time ↑	Women with excessive adipose tissue obscuring landmarks were excluded
Bertuit et al. (2015)	Longitudinal	Barefoot	Gaitrite	5–10 steps 9 gait trials	Velocity ↓, Stride Velocity ↓, Cadence ↓, Step Time ↓, Cycle Time ↓, Stride Length ↓, Step Length ↓, Step Width ↓	Socioeconomic status differences, Ignored speed and anthropometry
Carpes et al. (2008)	Longitudinal	Barefoot	3D motion analysis	Four walking cycles	Double Support Time (1st Trim ↓), Single Support (1st Trim ↑), Stance Phase (1st Trim ↓), Stride Length, Step Length	Lack pre-gestational evaluation
Forczek and Staszkiewicz (2012)	Longitudinal	Barefoot	3D motion analysis	On the floor 30 steps	Velocity↓, Frequency of Steps, Steps Length ↓	N/A
Wu et al. (2010)	Cross-sectional	N/A	3D motion analysis	Treadmill	Velocity ↓	N/A
Gilleard (2013)	Longitudinal	N/A	3D motion analysis	20 m Three trials	Velocity, Stride Length, Step Width	Small number of participants
Rothwell et al. (2020)	Longitudinal	Shoes	3D motion analysis	Treadmill	Step Width ↑	N/A

NP, Non-Pregnant, trim. = trimester, w.p. = week of pregnancy, m = month, postp. = *postpartum*, N/A = not available; ↓ = significant decrease (P < 0.05), ↑ = significant increase (P < 0.05).

### 2.4 Statistical analysis

Hedges’ g (adjusted standardized mean differences) was used to report effect sizes, allowing for precise comparisons across studies. Considering that the majority of the included studies had sample sizes between 6 and 70, Cohen’s d could potentially overestimate the true effect size in such cases. Therefore, Hedges’ g was chosen as it incorporates a correction factor for sample size, effectively reducing bias and providing a more reliable estimate.

To investigate the relationship between pregnancy stage and gait parameters, we first conducted a meta-analysis using the DerSimonian and Laird random-effects model with Knapp-Hartung adjustments, accounting for study heterogeneity. This method was chosen because it allows for the incorporation of both within-study and between-study variability. For studies with multiple intervention groups (e.g., different pregnancy stages), each group was analyzed separately to ensure that all relevant data were included without compromising the integrity of the results.

Following the meta-analysis, a continuous moderation analysis was performed for each gait parameter (e.g., gait speed, double support time), treating gestational weeks as a continuous moderator variable. Regression lines with 95% confidence intervals (CIs) were generated to visualize the relationship between pregnancy stages and gait changes. To ensure the robustness and reliability of the meta-regression models, each analysis required a minimum of 10 studies (Harrer et al., 2021).

All statistical analyses were conducted using R (version 3.6.2) with the ‘meta’ package (Schwarzer, 2007). Two-sided tests were applied, with a significance level set at P < 0.05. Publication bias was assessed using Egger’s test to detect any potential asymmetry in the data, ensuring the validity of our results.

## 3 Results

### 3.1 Participant characteristics and study designs

A total of 598 pregnant individuals from 15 countries were included in the analysis, with gait parameter data collected from 185 participants in the first trimester, 374 participants in the second trimester, and 482 participants in the third trimester. Some individuals were assessed at multiple stages of pregnancy, although some individuals were assessed at multiple stages, each stage’s data were analyzed as a separate group, ensuring that measurements from different trimesters were treated independently in the analysis. The participants’ ages ranged from 22 to 37 years, and their BMI values varied between 19.7 and 29.9 kg/m^2^. Five key gait parameters were identified for analysis: stride length, stride width, cycle time, double support time, and gait speed. Among the 21 included studies, 4 used early pregnancy (≤12 weeks) as the baseline for comparison, while 8 studies selected *postpartum* women as the control group. The remaining studies employed non-pregnant women who had never been pregnant as the control group. In cases where a non-pregnant control group was unavailable, gait parameters from 6 weeks *postpartum* or within 12 weeks of early pregnancy were used as control data. Given that gait changes are minimal during early pregnancy and physical function tends to partially recover by 6 weeks *postpartum* (Aguree and Gernand, 2019; Ramachandra, 2018; Conder et al., 2019), this approach is both reasonable and justifiable for approximating non-pregnant references. Most studies applied stringent exclusion criteria, typically excluding participants with orthopedic or neurological conditions, multiple pregnancies, high-risk pregnancies, or any factors that could affect gait.

Among the 21 studies evaluated, all were rated as high quality (≥75%), with scores ranging from 81.82% to 90.91%. The highest scores (90.91%) were achieved by Lymbery (Lymbery and Gilleard, 2005) and Bird (Bird et al., 1999), reflecting robust research designs and well-defined methodologies. Common strengths across these studies included clearly defined research questions, appropriate study designs, and comprehensive outcome measures. However, certain limitations were noted, such as the lack of random allocation and blinding of researchers and participants, which were often marked as “Not Applicable” (N/A). Despite these limitations, most studies provided detailed results and conclusions that were well-supported by their findings, thereby enhancing the overall reliability and validity of the evidence in this review.

Most of the studies (n = 16) employed a longitudinal design to observe gait changes over time in pregnant women, while 6 studies used a cross-sectional approach to capture gait characteristics at specific points during pregnancy or *postpartum*. The majority of participants (n = 12) walked barefoot, with only two studies using shoes, and 3D motion analysis emerged as the most common data collection method, employed in 13 studies. Walking protocols varied, with walkway lengths ranging from 5 to 20 m, while treadmill use was reported in only two studies. Frequently analyzed gait parameters included velocity, stride length, stride length, stance time, and double support time, with most studies observing a significant decrease in velocity and stride length during pregnancy.

### 3.2 Synthesis of results: meta-analysis

Random-effects meta-analyses were performed to assess different gait parameters across pregnancy. For stride length (k = 21), a modest yet significant effect size was identified (−0.29; 95% CI: 0.57 to −0.01; p = 0.04), accompanied by substantial heterogeneity (I^2^ = 80.80%). Step width (k = 16) demonstrated a moderate and significantly positive effect size (0.45; 95% CI: 0.25 to 0.66; p < 0.0001) with low heterogeneity (I^2^ = 18.89%). The analysis of gait speed (k = 23) indicated a moderate and significantly negative effect size (−0.55; 95% CI: 0.83 to −0.27; p = 0.0001), with considerable heterogeneity (I^2^ = 77.10%). Cycle time (k = 10) yielded a small to moderate positive effect size (0.38; 95% CI: 0.08 to 0.69; p = 0.01) with moderate heterogeneity (I^2^ = 53.73%). Finally, double support time (k = 13) indicated a small to moderate positive effect size (0.41; 95% CI: 0.02 to 0.79; p = 0.04) with substantial heterogeneity (I^2^ = 87.29%).

Egger’s test indicated no significant publication bias for gait cycle time (p = 0.74), gait speed (p = 0.94), stride width (p = 0.18), or double support time (p = 0.45), and stride length (p = 0.15). To assess the impact of pregnancy weeks on spatiotemporal gait characteristics, sensitivity analyses were performed on 5 gait parameters, including stride length, focusing on the effects of measurement devices and footwear type. The analyses showed no significant qualitative differences compared to the primary results, except for stride length, where variations in gait assessment tools and footwear did result in qualitative changes. In the first sensitivity analysis, which included only studies using 3D motion analysis technology, the results were as follows: double support time (F_1,7_ = 0.45, p = 0.21), gait cycle time (F_1,6_ = 0.92, p = 0.37), speed (F_1,16_ = 0.10, p = 0.75), and stride width (F_1,12_ = 0.046, p = 4.93). In the second analysis, focusing on barefoot conditions, the results were: double support time (F_1,7_ = 0.21, p = 0.65), speed (F_1,8_ = 0.001, p = 0.97), and stride width (F_1,7_ = 8.68, p = 0.02). These results indicate that double support time, cycle time, speed and stride width remained consistent across different gait assessment tools and footwear conditions. For stride length, sensitivity analyses revealed the following results: studies using 3D motion analysis technology showed (F_1,14_ = 3.45, p = 0.08), while studies involving barefoot conditions showed (F_1,11_ = 2.42, p = 0.14). The results suggest a variation from the primary analysis, implying that the choice of gait assessment tools and footwear could affect the association between pregnancy stages and stride length.

In studies using 3D motion analysis, no significant changes were observed for double support time, gait cycle time, speed, and stride width. For barefoot conditions, consistent findings were seen for double support time, speed, and stride width, except stride length, which demonstrated variation influenced by the assessment tools and footwear type.

**FIGURE 2:**
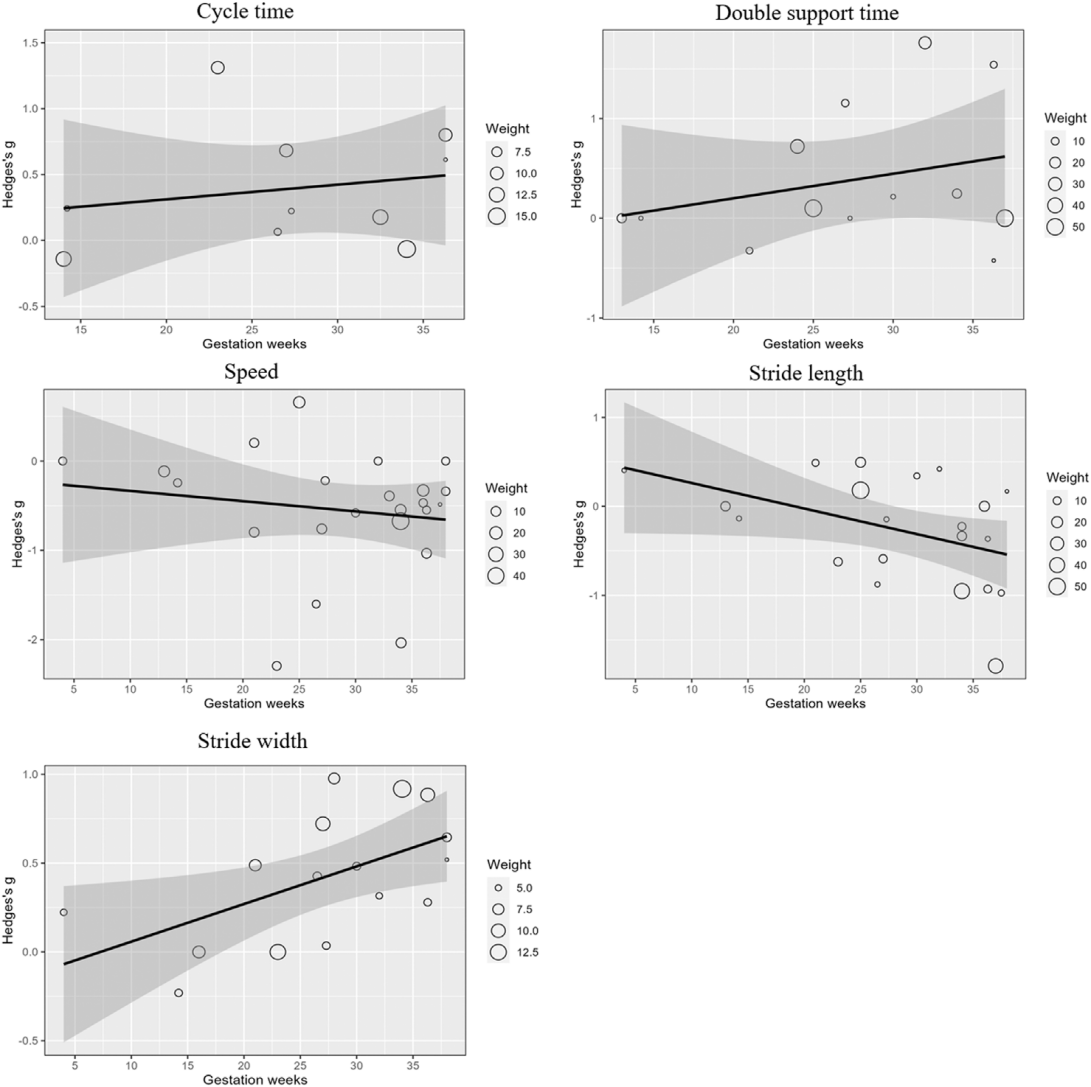
Meta-regression analysis of gait parameters across gestational weeks.

Meta-regression analyses examined the impact of gestational weeks on gait parameters, revealing that stride length (β = −0.03; 95% CI: 0.055 to −0.002; p < 0.05) significantly decreased, and stride width (β = 0.02; 95% CI: 0.003 to 0.039; p < 0.05) significantly increased as pregnancy progressed. No significant effects were observed for cycle time, double support time, or gait speed. These trends are visually represented in Figure 2, where the regression lines indicate the relationship between gestational weeks and the effect sizes for each gait parameter.

## 4 Discussion

This review identified a linear relationship between gestational age and specific spatiotemporal gait parameters. Notably, as pregnancy progresses, stride width increases and stride length decreases, suggesting these adjustments may help maintain balance and stability in response to physiological changes. By utilizing meta-regression to aggregate and analyze data across subgroups, this study addresses prior sample size limitations and provides a more comprehensive view of gait adaptations throughout pregnancy.

### 4.1 Cycle time

The meta-analysis demonstrated moderate heterogeneity (I^2^ = 53.73%), indicating variability in effect sizes across studies for gait cycle time. However, the meta-regression analysis revealed that gestational age accounted for only about 4.07% of this variability, suggesting that other factors, such as hormonal changes, balance adjustments, or biomechanical adaptations, might play a more significant role in influencing gait cycle time during pregnancy.

Five studies examined gait cycle time during pregnancy (Bertuit et al., 2015; Branco et al., 2013; Christensen et al., 2019; Gimunová et al., 2020; Branco et al., 2016), with two studies reporting a significant increase (Bertuit et al., 2015; Christensen et al., 2019). According to Christensen et al., pregnant women showed a 4% longer gait cycle time compared to non-pregnant women, with those experiencing pelvic girdle pain (PGP) displaying an even larger increase of 9% (Christensen et al., 2019). This suggests a potential association between PGP and increased gait cycle time, as pregnant women with PGP may adjust their gait to reduce discomfort or maintain stability. Similarly, Bertuit et al. reported a 10% increase in gait cycle time at a preferred walking speed during pregnancy (p = 0.003), attributing this change to an adaptive gait pattern aimed at enhancing safety and reducing the risk of falling (Bertuit et al., 2015). In contrast, the three other studies (Branco et al., 2013; Gimunová et al., 2020; Branco et al., 2016) did not report significant changes in gait cycle time during pregnancy. The lack of consistent findings across these studies indicates that the relationship between pregnancy, PGP, and gait cycle time may be more complex than initially thought, and warrants further investigation. These findings highlight that gait cycle time could be influenced by the presence of PGP, suggesting that pain management and stability considerations play a role in gait adaptations during pregnancy.

The discrepancies among these findings may be partly attributed to footwear. Sensitivity analysis indicated that being barefoot or wearing regular shoes did not significantly affect gait cycle time at different pregnancy stages, while specialized maternity footwear appeared to have a notable impact (Gimunová et al., 2020; Li et al., 2022). Gimunová et al. (2020) found that, although gait cycle time increased with pregnancy in a control group wearing ordinary shoes, this change was not significant. Conversely, women wearing specialized maternity shoes showed a significant increase at 28 and 37 weeks of pregnancy compared to 28 weeks after childbirth, suggesting that such footwear may help against the foot arches falling and influence gait patterns. This underscores the importance of considering footwear in gait analysis during pregnancy.

While gestational age alone cannot fully explain the changes in gait cycle time, the impact of PGP and specialized footwear indicates that pain management and external support play important roles in gait adaptations.

### 4.2 Double support time

The meta-analysis indicated substantial heterogeneity (I^2^ = 87.29%), suggesting considerable variability in effect sizes across studies examining double support time. However, the meta-regression analysis revealed that gestational weeks accounted for only a small fraction (7.98%) of this variability.

During pregnancy, women often adjust their gait to manage the forward shift in their center of gravity and increased body weight, enhancing gait stability (Carpes et al., 2008; Ramachandra, 2018; Błaszczyk et al., 2016; Maki, 1997; Cromwell and Newton, 2004). One common strategy is to extend the time their feet remain on the ground, which contributes to maintaining stability (Błaszczyk et al., 2016). Several studies have examined double support time during pregnancy (Foti et al., 2000; Carpes et al., 2008; Branco et al., 2013; Ramachandra, 2018; Krkeljas, 2018; Błaszczyk et al., 2016; Branco et al., 2016). Four studies identified a notable rise in double support time in pregnant women compared to their non-pregnant counterparts (Carpes et al., 2008; Branco et al., 2013; Ramachandra, 2018; Błaszczyk et al., 2016), whereas two studies observed no significant difference (Krkeljas, 2018; Branco et al., 2016). The meta-regression analysis revealed no significant linear association between double support time and the number of gestational weeks. This finding aligns with observations by Carpes et al., who reported that double support time is significantly shorter in both the second and third trimesters compared to pre-pregnancy. The most notable changes occurred between these trimesters, suggesting that these adjustments may happen more abruptly rather than gradually at specific stages of pregnancy (Carpes et al., 2008; Branco et al., 2013; Ramachandra, 2018). Such stage-specific changes may be influenced by rapid physiological and biomechanical adaptations, such as weight gain or shifts in the center of gravity, that occur as pregnancy progresses.

The walking protocols in these studies primarily involved short-distance indoor walking on firm surfaces, typically within 5–20 m, repeated in multiple trials. While these short-distance protocols are practical and feasible for laboratory settings, they may not fully reflect the natural walking patterns seen in longer, unbroken distances. This limitation is particularly relevant during pregnancy, as physiological changes, including reduced chest cavity space and potentially diminished cardiopulmonary capacity (LoMauro and Aliverti, 2015; Holmes et al., 2015; Sonaglioni et al., 2021), could alter gait characteristics over longer distances. Consequently, the findings from short-walk protocols may not capture gait adaptations that might occur in pregnant individuals during prolonged walking, where fatigue and cardiorespiratory demand might play a larger role. Further studies incorporating extended walking distances may provide a more comprehensive understanding of gait adaptations during pregnancy.

### 4.3 Gait velocity

The meta-analysis revealed a significant overall decrease in walking speed during pregnancy, but gestational weeks explained only a small portion (2.37%) of the variability, indicating that other factors might be more influential. While a general trend of decreasing walking speed was observed, this change did not follow a consistent linear pattern across pregnancy.

Twelve studies investigated walking speed during pregnancy (Zia et al., 2022; Foti et al., 2000; Bertuit et al., 2015; Forczek et al., 2020; Branco et al., 2013; Forczek and Staszkiewicz, 2012; Christensen et al., 2019; Krkeljas, 2018; Błaszczyk et al., 2016; Lymbery and Gilleard, 2005; Branco et al., 2016; Bagwell et al., 2020), with four reporting decreases, especially in the later stages (Zia et al., 2022; Bertuit et al., 2015; Forczek and Staszkiewicz, 2012; Błaszczyk et al., 2016). For instance, Christensen et al. (2019) found no significant change during mid-pregnancy, suggesting that gait alterations occur later. Bertuit et al. (2015) observed the lowest walking speed around the seventh month, followed by a slight increase toward term. These findings suggest that walking speed might change more at certain stages rather than gradually throughout pregnancy.

Differences in walking speed may be influenced by factors such as weight gain, psychological influences, or individual adaptations. While weight gain is often blamed for slower speeds, Blaszczyk’s study found that obese women walked faster than pregnant women, hinting at other factors like fear of movement or losing balance affecting gait (Bertuit et al., 2015; Błaszczyk et al., 2016; Gilleard, 2013). Gait velocity changes during pregnancy are complex, influenced by multiple factors beyond just gestational age. Future research should explore psychological, biomechanical, and individual factors to better understand and support gait adaptations in pregnant women.

### 4.4 Stride length

Research on stride length changes during pregnancy presents mixed findings. Some studies report no significant change (Foti et al., 2000; Forczek et al., 2020; Christensen et al., 2019; Krkeljas, 2018; Błaszczyk et al., 2016; Branco et al., 2016), while others indicate a decrease (Zia et al., 2022; Bertuit et al., 2015; Takeda et al., 2009; Branco et al., 2013). Our meta-analysis supports the latter, showing a statistically significant negative effect size of −0.29, with substantial heterogeneity (I^2^ = 80.80%), suggesting that stride length generally decreases during pregnancy, but with considerable variability across studies. To investigate this variability, we conducted a meta-regression analysis using gestational weeks as a moderator, which revealed gestational weeks as a significant predictor (p = 0.03), explaining approximately 24.2% of the variance in effect sizes. This analysis reduced overall heterogeneity from 80.80% to 61.24%, indicating that gestational weeks account for about 19.56% of the variability across studies, highlighting gestational age’s influence on stride length, which supports findings from earlier research (Zia et al., 2022; Bertuit et al., 2015; Takeda et al., 2009; Branco et al., 2013).

The observed reduction in stride length aligns with Jeanne et al., who reported a decrease ranging from 0.08 to 0.23 m during pregnancy (Bertuit et al., 2015). This change likely stems from mechanical factors such as increased pelvic width and anterior pelvic tilt (Foti et al., 2000), altering the body’s center of gravity as pregnancy progresses. Such adaptations aid in maintaining balance, as supported by Krkeljas, who found a significant correlation between shorter stride length and improved gait stability through reduced center of pressure and center of gravity tilt angles (Krkeljas, 2018). Our analysis suggests that as pregnancy progresses, a shorter stride length is adopted to enhance stability and reduce fall risk, particularly in the third trimester (Espy et al., 2010).

Despite gestational weeks being a significant factor explaining about 19.56% of the variability, a substantial portion of heterogeneity remains unexplained. The sensitivity analysis showed that when studies involving participants who wore shoes were excluded, the relationship between gestational weeks and stride length became weaker and was no longer statistically significant. This suggests that footwear plays a role in influencing stride length changes during pregnancy. Specifically, the presence of shoes may enhance or alter gait patterns, thereby affecting stride length measurements. Therefore, future research should carefully consider footwear conditions, and ideally, conduct separate analyses for barefoot and shoe-wearing participants. This approach would help to accurately assess the true impact of gestational weeks on stride length changes during pregnancy, accounting for the modifying effect of footwear.

### 4.5 Stride width

The meta-analysis showed a significant increase in stride width with low heterogeneity, indicating consistent findings across studies. The meta-regression analysis identified gestational weeks as a significant predictor of this effect, suggesting that stride width tends to increase as pregnancy progresses.

As pregnancy advances, a wider stride width aids in maintaining balance and minimizing the risk of falls. Krkeljas et al. found a significant correlation between changes in step width, stride length, and the COP/COG inclination angle, a key indicator of gait instability (Krkeljas, 2018). Moreover, this gait modification could serve as a strategy to alleviate pelvic pain by shifting the center of mass laterally, thereby lessening the strain on the hip abductors. This shift shortens the moment arm of the hip abductors, decreasing the force required to control pelvic lateral displacement, which can help alleviate pelvic pressure and pain (Neumann, 2010). Such adjustments enable pregnant women to maintain balance while minimizing discomfort in pelvic structures. However, Foti proposed that the increase in stride width might result from the natural widening of the pelvis during pregnancy, suggesting that this adjustment could be a mechanical consequence rather than purely a stability-driven adaptation (Foti et al., 2000). Therefore, whether the increase in stride width is primarily a response to stability needs or a result of pelvic changes remains unresolved. Nonetheless, the outcome is that each step involves more outward foot placement as pregnancy progresses.

Although we observed a linear relationship between stride width and gestational age, it remains unclear whether this increase is entirely due to stability needs or other physiological factors. Future research should explore the role of pelvic width changes and other factors in influencing gait patterns. Investigating additional contributors to stride width changes will help develop a more comprehensive model of gait adaptations during pregnancy.

## 5 Conclusion

This study provides a comprehensive analysis of gait adaptations during pregnancy by examining various spatiotemporal gait parameters. The meta-analysis and meta-regression revealed significant linear relationships between gestational age and certain gait adjustments, specifically an increase in stride width and a decrease in stride length as pregnancy progresses. These findings suggest that pregnant women adopt specific gait modifications to maintain balance and stability in response to physiological changes such as weight gain, shifts in the center of gravity, and pelvic adaptations.

The increase in stride width appears to be a consistent adjustment aimed at enhancing stability and reducing the risk of falls. However, this change may result not only from a need for increased stability but also from mechanical changes, such as pelvic widening, driven by hormonal adaptations. Future studies might explore whether stride width adjustments are primarily influenced by structural pelvic changes or balance requirements. The decrease in stride length is likely a compensatory mechanism to improve gait stability, particularly in the later stages of pregnancy. The influence of footwear was also identified as a significant factor affecting stride length, highlighting the importance of considering external support in gait analysis during pregnancy.

In addition, hormonal factors, such as relaxin and estrogen, which increase joint flexibility during pregnancy, may also play a role in gait changes. Psychological influences, like increased anxiety or fear of falling, may further modify gait parameters, contributing to adjustments like shorter stride length or slower gait speed. Other gait parameters, such as gait cycle time and gait velocity, showed variability across studies and did not exhibit a consistent linear relationship with gestational age. Factors like pelvic girdle pain (PGP), psychological influences, and individual adaptations may play more significant roles in these parameters than gestational progression alone. The presence of PGP was associated with increased gait cycle time, suggesting that pain management is crucial for understanding and supporting gait adaptations.

The substantial heterogeneity observed in some gait parameters indicates that multiple factors contribute to gait changes during pregnancy. To address this, future research should employ standardized methodologies, such as consistent footwear protocols and uniform gait measurement techniques, to reduce extraneous variability and improve comparability across studies. This approach would allow for a more precise understanding of gait adaptations during pregnancy and help in identifying specific variables, such as psychological influences and individual physical characteristics, that warrant further investigation.

In conclusion, this study underscores the complexity of gait adaptations during pregnancy, emphasizing that while gestational age influences certain gait parameters, a multitude of factors contribute to these changes. Practical applications of these findings could include incorporating routine gait assessments into prenatal care, helping healthcare providers monitor and address changes in gait that may affect maternal mobility and safety. Recognizing and addressing these factors is essential for supporting the health and wellbeing of pregnant women through evidence-based guidelines and interventions.

## References

[B1] Aguree S. Gernand A. D. (2019). Plasma volume expansion across healthy pregnancy: a systematic review and meta-analysis of longitudinal studies. BMC Pregnancy Childbirth 19, 508–511. 10.1186/s12884-019-2619-6 31856759 PMC6924087

[B2] Atay E. Iz F. B. (2015). Investigation of the effect of changes in muscle strength in gestational ageupon fear of falling and quality of life. Turkish J. Med. Sci. 45 (4), 977–983. 10.3906/sag-1404-9 26422877

[B3] Bagwell J. J. Reynolds N. Walaszek M. Runez H. Lam K. Smith J. A. (2020). Lower extremity kinetics and muscle activation during gait are significantly different during and after pregnancy compared to nulliparous females. Gait Posture 81, 33–40. 10.1016/j.gaitpost.2020.07.002 32659459

[B4] Bertuit J. Feipel V. Rooze M. (2015). Temporal and spatial parameters of gait during pregnancy. Acta Bioeng. biomechanics 17 (2), 93–101. 10.5277/ABB-00092-2014-03 26399272

[B5] Bird A. R. Menz H. B. Hyde C. C. (1999). The effect of pregnancy on footprint parameters. A prospective investigation. J. Am. Podiatr. Med. Assoc. 89 (8), 405–409. 10.7547/87507315-89-8-405 10466293

[B6] Bjelica A. Cetkovic N. Trninic-Pjevic A. Mladenovic-Segedi L. (2018). The phenomenon of pregnancy—a psychological view. Ginekol. Pol. 89 (2), 102–106. 10.5603/gp.a2018.0017 29512815

[B7] Błaszczyk J. W. Opala-Berdzik A. Plewa M. (2016). Adaptive changes in spatiotemporal gait characteristics in women during pregnancy. Gait Posture 43, 160–164. 10.1016/j.gaitpost.2015.09.016 26480840

[B8] Branco M. Santos-Rocha R. Aguiar L. Vieira F. Veloso A. (2013). Kinematic analysis of gait in the second and third trimesters of pregnancy. J. Pregnancy 2013 (1), 718095. 10.1155/2013/718095 23431450 PMC3572696

[B9] Branco M. Santos-Rocha R. Aguiar L. Vieira F. Veloso A. P. (2022). “Biomechanical adaptations of gait in pregnancy: implications for physical activity and exercise,” in Exercise and physical activity during pregnancy and postpartum: evidence-based guidelines. Springer, 105–153. 10.1007/978-3-319-91032-1_5

[B10] Branco M. A. Santos-Rocha R. Vieira F. Aguiar R. Veloso A. P. (2016). Three-dimensional kinematic adaptations of gait throughout pregnancy and post-partum. Acta Bioeng. Biomechanics 18 (2), 153-–162. 10.5277/ABB-00418-2015-05 27406315

[B11] Carpes F. Griebeler D. Kleinpaul J. Mann L. Mota C. (2008). Women able-bodied gait kinematics during and post pregnancy period. Rev. Bras. Biomecânica 9 (16).

[B12] Christensen L. Veierød M. B. Vøllestad N. K. Jakobsen V. E. Stuge B. Cabri J. (2019). Kinematic and spatiotemporal gait characteristics in pregnant women with pelvic girdle pain, asymptomatic pregnant and non-pregnant women. Clin. Biomech. 68, 45–52. 10.1016/j.clinbiomech.2019.05.030 31158589

[B13] Conder R. Zamani R. Akrami M. (2019). The biomechanics of pregnancy: a systematic review. J. Funct. Morphol. Kinesiol. 4 (4), 72. 10.3390/jfmk4040072 33467386 PMC7739277

[B14] Cromwell R. L. Newton R. A. (2004). Relationship between balance and gait stability in healthy older adults. J. Aging Phys. Act. 12 (1), 90–100. 10.1123/japa.12.1.90 15211023

[B15] ElDeeb A. M. Hamada H. A. Abdel-Aziem A. A. Youssef A. M. (2016). The relationship between trunk and pelvis kinematics during pregnancy trimesters. Acta Bioeng. Biomechanics 18 (4), 79–85. 10.5277/ABB-00544-2016-03 28133373

[B16] Espy D. Yang F. Pai Y.-C. (2010). Control of center of mass motion state through cuing and decoupling of spontaneous gait parameters in level walking. J. Biomech. 43 (13), 2548–2553. 10.1016/j.jbiomech.2010.05.015 20542513 PMC2942766

[B17] Feldt–Rasmussen U. Mathiesen E. R. (2011). Endocrine disorders in pregnancy: physiological and hormonal aspects of pregnancy. Best Pract. and Res. Clin. Endocrinol. and metabolism 25 (6), 875–884. 10.1016/j.beem.2011.07.004 22115163

[B18] Forczek W. Ivanenko Y. Curyło M. Frączek B. Masłoń A. Salamaga M. (2019). Progressive changes in walking kinematics throughout pregnancy—a follow up study. Gait Posture 68, 518–524. 10.1016/j.gaitpost.2019.01.004 30623846

[B19] Forczek W. Ivanenko Y. Salamaga M. Sylos-Labini F. Frączek B. Masłoń A. (2020). Pelvic movements during walking throughout gestation-the relationship between morphology and kinematic parameters. Clin. Biomech. 71, 146–151. 10.1016/j.clinbiomech.2019.11.001 31743885

[B20] Forczek W. Ivanenko Y. P. Bielatowicz J. Waclawik K. (2018). Gait assessment of the expectant mothers - systematic review. Gait Posture 62, 7–19. 10.1016/j.gaitpost.2018.02.024 29500941

[B21] Forczek W. Staszkiewicz R. (2012). Changes of kinematic gait parameters due to pregnancy. Acta Bioeng. Biomech. 14 (4), 113–119. 10.1016/j.gaitpost.2019.01.004 23394129

[B22] Foti T. Davids J. R. Bagley A. (2000). A biomechanical analysis of gait during pregnancy. JBJS 82 (5), 625–632. 10.2106/00004623-200005000-00003 10819273

[B23] Fukan M. Nomura Y. Tsukahara Y. (2024). Does the pregnancy-related adaptation of gait biomechanics after childbirth recover to its pre-pregnancy state? systematic Review. Gait Posture. 10.1016/j.gaitpost.2024.03.011 38569400

[B24] Gilleard W. L. (2013). Trunk motion and gait characteristics of pregnant women when walking: report of a longitudinal study with a control group. BMC Pregnancy Childbirth 13, 71–78. 10.1186/1471-2393-13-71 23514204 PMC3614455

[B25] Gimunová M. Zvonař M. Sebera M. Turčínek P. Kolářová K. (2020). Special footwear designed for pregnant women and its effect on kinematic gait parameters during pregnancy and postpartum period. PLoS One 15 (5), e0232901. 10.1371/journal.pone.0232901 32396578 PMC7217473

[B26] Harrer M. Cuijpers P. Furukawa T. Ebert D. (2021). Doing meta-analysis with R: a hands-on guide. Chapman and Hall/CRC.

[B27] Holmes S. Kirkpatrick I. D. Zelop C. M. Jassal D. S. (2015). MRI evaluation of maternal cardiac displacement in pregnancy: implications for cardiopulmonary resuscitation. Am. J. Obstet. Gynecol. 213 (3), 401. e1–e401.e5. 10.1016/j.ajog.2015.05.018 25981849

[B28] Ibrahim S. M. Nicoloro-SantaBarbara J. Auerbach M. V. Rosenthal L. Kocis C. Busso C. E. (2019). Pregnancy-specific coping and changes in emotional distress from mid-to late pregnancy. J. Reprod. Infant Psychol. 37 (4), 397–412. 10.1080/02646838.2019.1578871 30773900

[B29] Kazma J. M. van den Anker J. Allegaert K. Dallmann A. Ahmadzia H. K. (2020). Anatomical and physiological alterations of pregnancy. J. Pharmacokinet. Pharmacodyn. 47 (4), 271–285. 10.1007/s10928-020-09677-1 32026239 PMC7416543

[B30] Kerbourc’h F. Bertuit J. Feipel V. Rooze M. (2017). Pregnancy and pelvic girdle pain: analysis of center of pressure during gait. J. Am. Podiatr. Med. Assoc. 107 (4), 299–306. 10.7547/15-087 28880594

[B31] Kmet L. M. Cook L. S. Lee R. C. Standard quality assessment criteria for evaluating primary research papers from a variety of fields. 2004.

[B32] Krkeljas Z. (2018). Changes in gait and posture as factors of dynamic stability during walking in pregnancy. Hum. Mov. Sci. 58, 315–320. 10.1016/j.humov.2017.12.011 29254847

[B33] Landis J. R. Koch G. G. (1977). The measurement of observer agreement for categorical data. Biometrics 33, 159–174. 10.2307/2529310 843571

[B34] Li X. Lu Z. Sun D. Xuan R. Zheng Z. Gu Y. (2022). The influence of a shoe’s heel-toe drop on gait parameters during the third trimester of pregnancy. Bioengineering 9 (6), 241. 10.3390/bioengineering9060241 35735484 PMC9220068

[B35] LoMauro A. Aliverti A. (2015). Respiratory physiology of pregnancy: physiology masterclass. Breathe 11 (4), 297–301. 10.1183/20734735.008615 27066123 PMC4818213

[B36] Lymbery J. K. Gilleard W. (2005). The stance phase of walking during late pregnancy: temporospatial and ground reaction force variables. J. Am. Podiatr. Med. Assoc. 95 (3), 247–253. 10.7547/0950247 15901811

[B37] Maki B. E. (1997). Gait changes in older adults: predictors of falls or indicators of fear? J. Am. Geriatr. Soc. 45 (3), 313–320. 10.1111/j.1532-5415.1997.tb00946.x 9063277

[B38] McCrory J. L. Chambers A. J. Daftary A. Redfern M. S. (2014). Ground reaction forces during stair locomotion in pregnant fallers and non-fallers. Clin. Biomech. 29 (2), 143–148. 10.1016/j.clinbiomech.2013.11.020 24359627

[B39] Mei Q. Gu Y. Fernandez J. (2018). Alterations of pregnant gait during pregnancy and post-partum. Sci. Rep. 8 (1), 2217–7. 10.1038/s41598-018-20648-y 29396468 PMC5797072

[B40] Moher D. Shamseer L. Clarke M. Ghersi D. Liberati A. Petticrew M. (2015). Preferred reporting items for systematic review and meta-analysis protocols (PRISMA-P) 2015 statement. Syst. Rev. 4, 1–9. 10.1186/2046-4053-4-1 25554246 PMC4320440

[B41] Murray I. Hendley J. (2020). Change and adaptation in pregnancy. Myles' Textb. Midwives E-Book, 197.

[B42] Nascimento S. L. Surita F. G. Godoy A. C. Kasawara K. T. Morais S. S. (2015). Physical activity patterns and factors related to exercise during pregnancy: a cross sectional study. PLoS One 10 (6), e0128953. 10.1371/journal.pone.0128953 26083416 PMC4470997

[B43] Neumann D. A. (2010). Kinesiology of the hip: a focus on muscular actions. J. Orthop. Sports Phys. Ther. 40 (2), 82–94. 10.2519/jospt.2010.3025 20118525

[B44] Page M. J. McKenzie J. E. Bossuyt P. M. Boutron I. Hoffmann T. C. Mulrow C. D. (2021). The PRISMA 2020 statement: an updated guideline for reporting systematic reviews. BMJ 372, n71. 10.1136/bmj.n71 33782057 PMC8005924

[B45] Ramachandra P. (2018). Spatio-temporal gait parameters during pregnancy and postpartum. Online J. Health Allied Sci. 17 (1).

[B46] Rothwell S. A. Eckland C. B. Campbell N. Connolly C. P. Catena R. D. (2020). An analysis of postpartum walking balance and the correlations to anthropometry. Gait Posture 76, 270–276. 10.1016/j.gaitpost.2019.12.017 31883494

[B47] Schwarzer G. (2007). meta: an R package for meta-analysis. R. news 7 (3), 40–45.

[B48] Sonaglioni A. Esposito V. Caruso C. Nicolosi G. L. Bianchi S. Lombardo M. (2021). Chest conformation spuriously influences strain parameters of myocardial contractile function in healthy pregnant women. J. Cardiovasc Med. 22 (10), 767–779. 10.2459/jcm.0000000000001213 34487054

[B49] Stroup D. F. Berlin J. A. Morton S. C. Olkin I. Williamson G. D. Rennie D. (2000). Meta-analysis of observational studies in epidemiology: a proposal for reporting. JAMA 283 (15), 2008–2012. 10.1001/jama.283.15.2008 10789670

[B50] Sunaga Y. Anan M. Shinkoda K. (2013). Biomechanics of rising from a chair and walking in pregnant women. Appl. Ergon. 44 (5), 792–798. 10.1016/j.apergo.2013.01.010 23452381

[B51] Takeda K. Junji K. Aya T. Sigeko F. Yosie E. (2009). P959 an analysis of gait in the third trimester of pregnancy–gait analysis of the single support phase in the frontal plane. Int. J. Gynecol. and Obstetrics 107, S683–S. 10.1016/s0020-7292(09)62446-2

[B52] Wong J. K. McGregor A. H. (2018). Spatiotemporal gait changes in healthy pregnant women and women with pelvic girdle pain: a systematic review. J. Back Musculoskelet. Rehabil. 31 (5), 821–838. 10.3233/bmr-170828 29865027

[B53] Wu W.-h. Onno G. M. Huang Z.-j. Liu X.-x. (2010). Motor coordination mode of transverse pelvic and thoracic rotations during walking in healthy pregnant women. Chin. J. Tissue Eng. Res. 14 (2), 375. 10.3969/j.issn.1673-8225.2010.02.043

[B54] Zia A. Sheraz S. Asif R. Khalid A. Razzaq A. (2022). Biomechanical changes in third trimester of pregnant females in comparison to non pregnant females of same age group. J. Med. Sci. 30 (1), 28–31. 10.52764/jms.22.30.1.6

