# Supplementary material for: Pregnancy-induced gait alterations: meta-regression evidence of spatiotemporal adjustments
Source: Front Bioeng Biotechnol. 2024 Dec 17;12:1506002. doi: 10.3389/fbioe.2024.1506002 (PMC11685021; doi:10.3389/fbioe.2024.1506002)
Supplement: Supplementary file 1 [file Table1.docx]

Appendix 1: Search strategies were used within five databases.

| Pregnancy | Gait | Spatiotemporal |
| --- | --- | --- |
| Pregnant | Walk | Step and time |
| Maternal | Locomotion | spatio-temporal |
| Gestation | Walking | Spatia and temporal |
| Antenatal | stride | parameter |
| Prenatal | ambulation | Parameters |
| gravidity |  |  |
| Expectant mother |  |  |
| Expecting |  |  |
|  |  |  |

| Database | Search terms |
| --- | --- |
| Pubmed  1981-2023 | (("Pregnancy" OR "Pregnant women" OR "Maternal" OR "Gestation" OR "Antenatal" OR "Prenatal" OR "gravidity" OR "Expectant mother" OR "Expecting") AND ("Gait" OR "Walking" OR "Locomotion" OR "ambulation" OR "stride") AND ("Spatiotemporal" OR "(step AND time)" OR "spatio-temporal" OR "spatial-temporal" OR "parameter" OR "Parameters")) |
| Web of science | TS=((Pregnancy OR "Pregnant women" OR Maternal OR Gestation OR Antenatal OR Prenatal OR gravidity OR "Expectant mother" OR Expecting) AND (Gait OR Walking OR Locomotion OR ambulation OR stride) AND (Spatiotemporal OR (step and time) OR spatio-temporal OR spatial-temporal OR parameter OR Parameters)) |
| Scopus | TITLE-ABS-KEY((Pregnancy OR "Pregnant women" OR Maternal OR Gestation OR Antenatal OR Prenatal OR gravidity OR "Expectant mother" OR Expecting) AND (Gait OR Walking OR Locomotion OR ambulation OR stride) AND (Spatiotemporal OR (step AND time) OR spatio-temporal OR spatial-temporal OR parameter OR Parameters)) |
| EBSCO | ("Pregnancy" OR "Pregnant women" OR "Maternal" OR "Gestation" OR "Antenatal" OR "Prenatal" OR "gravidity" OR "Expectant mother" OR "Expecting") AND ("Gait" OR "Walking" OR "Locomotion" OR "ambulation" OR "stride") AND ("Spatiotemporal" OR "Step and Time" OR "spatio-temporal" OR "spatial-temporal" OR "parameter" OR "Parameters") |
| Embase | (pregnancy OR "pregnant women" OR maternal OR gestation OR antenatal OR prenatal OR gravidity OR "expectant mother" OR expecting) AND (gait OR walking OR locomotion OR ambulation OR stride) AND (spatiotemporal OR "step and time" OR spatio-temporal OR spatial-temporal OR parameter OR parameters) |
| Cochrane | (Pregnancy OR Pregnant women OR Maternal OR Gestation OR Antenatal OR Prenatal OR Gravidity OR Expectant mother OR Expecting) AND (Gait OR Walking OR Locomotion OR Ambulation OR Stride) AND (Spatiotemporal phenomenon OR Time Factors OR Spatial Behavior OR Movement) |
